# The Impact of Negative Emotional Dysregulation on the Outcome of Bariatric Surgery in Patients with Severe Obesity: An Observational One-Year Follow-Up Study

**DOI:** 10.3390/jcm13175158

**Published:** 2024-08-30

**Authors:** Margherita Barbuti, Giulia D’Alessandro, Francesco Weiss, Alba Calderone, Ferruccio Santini, Giulio Perugi, Icro Maremmani

**Affiliations:** 1 2nd Psychiatry Unit, Department of Clinical and Experimental Medicine, University Hospital of Pisa, Via Roma 67, 56126 Pisa, Italy; margherita.barbuti@gmail.com (M.B.); giulia.dalessandro26@gmail.com (G.D.); francesco_weiss@libero.it (F.W.); giulio.perugi@med.unipi.it (G.P.); 2 1st Endocrinology Unit, Department of Clinical and Experimental Medicine, Obesity and Lipodystrophy Research Center, University Hospital of Pisa, Via Paradisa 2, 56124 Pisa, Italy; albacalderone@libero.it (A.C.); ferruccio.santini@med.unipi.it (F.S.); 3 Saint Camillus International University of Health and Medical Sciences (UniCamillus), 00131 Rome, Italy; 4 G. De Lisio Institute of Behavioral Sciences, 56127 Pisa, Italy

**Keywords:** obesity, bariatric surgery, weight loss, mood disorders, binge-eating disorder, emotional dysregulation

## Abstract

**Background**: Psychiatric symptoms are highly prevalent in patients with severe obesity, often representing pivotal factors in the development and progression of this condition. This study examines the association between negative emotional dysregulation (NED) and weight loss following bariatric surgery. **Methods**: Ninety-nine patients were consecutively enrolled at the Obesity Center of the Pisa University Hospital between March 2019 and February 2021, during a routine psychiatric evaluation before bariatric surgery. Psychopathological dimensions were assessed using the Mini-International Neuropsychiatric Interview (MINI), the Reactivity, Intensity, Polarity, and Stability questionnaire in its 40-item version (RIPoSt-40), the Wender–Reimherr Adult Attention Deficit Disorder Scale (WRAADDS) and the Barratt Impulsiveness Scale (BIS-11). Based on a RIPoSt-40 cut-off score of 70, subjects were divided into two groups: with (NED+) and without (NED−) NED. **Results**: NED+ subjects had a higher rate of psychiatric comorbidities and eating disorders than NED− patients. Of the total sample, 76 underwent bariatric surgery, and 65 of them were re-evaluated one-year after surgery. Among them, 10 of 28 NED+ subjects (37.5%) had inadequate weight loss one year after surgery compared to 5 of 37 NED− subjects (13.5%) (*p* = 0.035, OR 3.55, 95%, C.I. 1.05–12.03). **Conclusions**: Our results suggest a significant association between NED and inadequate weight loss at one-year post surgery.

## 1. Introduction

Obesity is a heterogeneous condition, representing the final common pathway of a variety of predisposing factors and primary disorders. Currently, it is a highly prevalent condition that represents a major concern for healthcare systems throughout the world [1,2]. In high-income countries, obesity is common among men and women of all ages but disproportionately affects disadvantaged groups [3,4]. In low-income countries, obesity is more prevalent among middle-aged adults, especially women, from affluent urban backgrounds [3,4]. Overall, women have a higher prevalence of obesity than men at all sociodemographic levels [3].

The problem of obesity is associated with a heterogeneous number of environmental factors. In western countries the large availability of highly palatable food with poor nutritional properties, but high content in calories, undoubtedly facilitates the development of the condition, particularly in those regions where junk food is much more affordable than a healthy and balanced nutrition [5]. Furthermore, people are often exposed to the contradictory drives of a capitalistic marketing that is equally aggressive in promoting unhealthy foodstuffs and beverages and fostering an endless quest for thinness, which implies selling diets, diet pills, and all sorts of variably legitimate remedies [5,6]. While in the developed world the preference for leanness is often mirrored by a negative attitude towards different bodily states (a phenomenon referred to as “fat phobia” or “adipophobia”), in other socio-cultural contexts (namely in northern Africa and the Middle East) being overweight or obese is often considered desirable, either in terms of a fertility-related standard of attractiveness or as a marker of socioeconomic well-being [7,8].

It is increasingly evident that considering obesity simplistically as an imbalance between caloric intake and energy expenditure is insufficient to explain the poor effectiveness of treatments proposed through behavioral changes aimed at reducing energy intake and increasing physical exercise [5]. Psychiatric disorders and neurodevelopmental trait variables related to affective, cognitive, and behavioral regulation have a tremendous impact on eating behaviors and weight trajectories, yet they are often neglected by the clinicians, overshadowed by approaches based solely on caloric deficit and physical activity [9].

Considering that psychiatric disorders, such as binge-eating disorder, mood disorders, panic disorder, attention-deficit/hyperactivity disorder (ADHD), are extremely common in obese patients, treating obesity without a personalized and multidimensional strategy often leads to unsatisfactory results, sometimes even compounding the initial pathology and triggering disease progression [10].

ADHD is the most common neurodevelopmental disorder and is characterized by core symptoms of inattention, hyperactivity, and impulsivity [11]. Diagnosis usually occurs in childhood, but at least 60–70% of children with ADHD still have symptoms as adults [12]. The extreme instability and emotional reactivity, defined as emotional dysregulation, represent a crucial aspect of adult ADHD [13,14]. Similar neurobiological and neuropsychological abnormalities, such as disruptions in reward pathways, executive function, and emotion regulation, have been found to underlie both obesity and ADHD [15]. This translates into altered eating habits and difficulties in making lasting changes in diet and lifestyle [16].

Bariatric surgery, although leading to significant weight reduction and improved quality of life in a substantial proportion of patients, requires specific changes in diet and lifestyle that not all subjects manage to observe in the long term. This appears to be particularly true for patients with a more unfavorable psychopathological and neurocognitive profile [17]. In fact, psychiatric disorders require specialized preoperative evaluation in candidates to bariatric surgery, albeit they do not represent an absolute contraindication to this approach, and every case should be assessed individually [10].

Failure to diagnose and manage psychiatric disorders and symptoms in the evaluation of obese individuals can have a significant clinical impact on the long-term outcome of bariatric surgery. In fact, psychiatric disorders associated with obesity, such as depression, anxiety, and eating disorders, may compromise the effectiveness of treatment and lead to less favorable outcomes, including an increased risk of weight regain and medical complications [18,19]. In addition, bariatric surgery may be less effective if underlying psychopathological factors are not addressed, thus compromising the sustainability of benefits over time [20]. Failure to address these issues may also lead to increased healthcare costs, as patients may require more intensive and frequent care. Future guidelines should focus on the psychiatric and psychopathological aspects to be fully evaluated before and after bariatric surgery and the treatments to be implemented [21].

The purpose of this study is to examine the clinical differences between obese bariatric patients with high and low negative emotional dysregulation (NED) and to evaluate the relationship of this construct with clinical outcomes after bariatric surgery. Our working hypothesis is that high NED is associated with lower long-term weight loss after surgical treatment.

## 2. Materials and Methods

### 2.1. Sample and Study Design

This is a post hoc analysis of a prospective, observational study [22], in which 99 adult participants with obesity and seeking bariatric surgery were consecutively enrolled between March 2019 and February 2021 at the Obesity Center of the 1st Endocrinology Unit of the Pisa University Hospital. The response rate was 94.3% (99 of 105 patients offered the study). The sample size was smaller than expected due to the COVID-19 pandemic, which caused the suspension of pre-bariatric evaluations at our Obesity Center for a few months in 2020. Subjects were recruited during routine psychiatric evaluation before bariatric surgery. Inclusion criteria were age ≥ 18 years, class III obesity (BMI ≥ 40 kg/m^2^) or class II obesity (BMI ≥ 35 kg/m^2^), plus an obesity-related medical comorbidity. Patients who were unable to complete the self-report questionnaires or who had unstable and/or severe medical or psychiatric conditions were excluded from recruitment. Participants who underwent bariatric surgery were subsequently monitored for weight- and obesity-related comorbidities at follow-up visits at 1 month (±1 week), 3 months (±1 week), 7 months (±2 weeks), and 12 months (±2 weeks) after surgery. To detect a minimal clinically important difference in excess BMI loss (30%) between NED+ and NED− patients at 1-year follow-up after bariatric surgery, we calculated that a sample size of at least 70 participants (35 per group) would be required, with 80% power at the 5% significance level.

### 2.2. Data Collection and Clinical Assessment

At baseline, before the bariatric intervention, the participating psychiatrists collected sociodemographic data (age, sex, marital status, education, employment status) and several clinical variables (e.g., psychophysical development, familial history of psychiatric disorders, previous and current psychotropic treatments). Current and/or lifetime psychiatric comorbidity, according to Diagnostic and Statistical Manual of Mental Disorders, 5th edition (DSM-5), criteria, was assessed using the Mini-International Neuropsychiatric Interview (MINI, version 7.0.2.), a brief, structured diagnostic interview designed to meet the need for brief but accurate psychiatric assessment [23].

During the baseline psychiatric evaluation, the Wender–Reimherr Adult Attention Deficit Disorder Scale (WRAADDS), a clinician-rated scale based on the Utah Criteria for adult ADHD, was administered to assess the severity of ADHD symptoms in 7 domains (attentional difficulties, persistent motor hyperactivity, hot temper, affective lability, emotional over-reactivity, disorganization, and impulsivity) [24]. Each domain is rated from 0 to 4 (none, mild, moderate, quite a bit, or very much). Factor analysis of the WRAADDS supports the validity of two types of ADHD in adults: the inattentive presentation (WRAADDS-I, which includes the attention difficulties and disorganization domains) and the emotional dysregulation presentation (WRAADDS-ED, which includes the temper, affective lability, and emotional over-reactivity domains), with hyperactivity and impulsivity common to both types [25].

Participants were also administered the Reactivity, Intensity, Polarity, and Stability self-questionnaire in its validated 40-item version (RIPoSt-40). Items rated on a Likert scale ranging from 1 (“never”) to 6 (“always”) are summed to yield four subscale scores assessing affective instability, positive and negative emotionality, and emotional impulsivity, and a second-order negative emotional dysregulation (NED) score consisting of the affective instability, negative emotionality, and impulsivity subscales. RIPoSt-40 subscales have proven good test–retest reliability and high internal consistency in both clinical and non-clinical samples [26]. Subjects were divided into two groups based on the presence of greater or lesser negative emotional dysregulation according to the median NED score of the RIPoSt-40, considering a cut-off of ≥70.

Finally, the Barratt Impulsiveness Scale, 11th edition (BIS-11) is composed of six first-order components (attention, cognitive instability, motor impulsiveness, perseverance, cognitive complexity and self-control) combined into three second-order factors representing three types of impulsivity: attentional impulsiveness (attention + cognitive instability), motor impulsiveness (motor + perseverance) and non-planning impulsiveness (cognitive complexity + self-control). It is a 30-item self-report questionnaire rated on a four-point Likert scale from 1 = rarely/never to 4 = almost always/always [27].

For participants who underwent surgery, information on BMI at the time of surgery and type of surgery was obtained from medical records. Subsequently, BMI was measured at each follow-up visit. Due to the coronavirus disease 2019 (COVID-19) pandemic, some of the follow-up assessments were conducted by teleconsultation.

For patients who completed the 1-year follow-up, inadequate excess BMI loss at 12 months after the intervention was defined as [(BMI at baseline − BMI at 12-month follow-up)/(BMI at baseline − 25) × 100], less than 50%.

### 2.3. Statistical Analysis

Descriptive statistics were used to summarize sample characteristics, reported as means and standard deviations (SDs) for continuous variables with a normal distribution and as numbers and percentages for categorial variables. The Shapiro–Wilk test was used to test the normality of continuous variables. Comparisons between subjects with and without NED were made using the chi-square test for categorical variables and Student’s *t*-test for continuous variables. Statistical significance was set at *p* < 0.05 (2-tailed).

Given the large number of comparisons and small sample size, we added the Benjamini–Hochberg correction for statistical significance in univariate comparisons. A logistic regression model was performed to determine the predictive value of clinical characteristics on the presence of major NED. Statistical significance after Benjamini–Hochberg correction in univariate comparisons was initially used as a threshold for inclusion of a variable in the regression model. We then removed variables (mood disorders, BIS total score, and WRAADDS total score) that were dependent on others included in the model (bipolar disorder, BIS subscale score, WRAADDS-ED score). For the calculation of ORs of categorical variables referring to psychiatric comorbidities (BED, bipolar disorder, etc.) we used the absence of the above comorbidity (non-BED, non-bipolar disorder, etc.) as the reference category. We used the statistical routines of IBM SPSS Statistics for Mac, version 25.0 (SPSS Inc., Chicago, IL, USA).

## 3. Results

### 3.1. Clinical and Sociodemographic Characteristics of the Total Sample (Table 1)

Female participants made up almost three quarters of the sample (71.7%). The mean age of the total sample was approximately 46 years (range 21–68), with a normal distribution. Of the total sample, 20% were single at the time of assessment, 65% were married, and 15% were divorced or widowed. According to the International Standard Classification of Education (ISCED), half of the subjects had completed upper secondary education, about one-third had completed lower secondary education, and a minority had a tertiary education degree. Regarding employment status, most patients (66%) were employed, 28% were unemployed, and only six patients were retired or disabled.

**Table 1:** Clinical and sociodemographic characteristics of the total sample (*n* = 99) at baseline assessment.

Age (mean, sd)	45.83 (11.23)
Female sex (*n*, %)	71 (71.7%)
BMI, Kg/m^2^ (mean, sd)	44.94 (6.52)
Marital status (*n*, %)	
Single	20 (20.2%)
Married	64 (64.6%)
Divorced/Widowed	15 (15.2%)
Education (*n*, %)	
Degree	12 (12.1%)
High school diploma	50 (50.5%)
Secondary/Elementary school diploma	37 (37.4%)
Work status (*n*, %)	
Unemployed	28 (28.3%)
Employed	65 (65.7%)
Disabled/Retired	6 (6.1%)
Lifetime psychiatric comorbidity (*n*, %)	
Mood disorders	60 (60.6%)
Eating disorders	55 (55.6%)
Anxiety disorder	46 (46.5%)

The mean BMI was 44.9 kg/m^2^ (range 34.6–65.8), and 74% of participants had a BMI ≥ 40 kg/m^2^. The mean age of reported obesity onset was 17 years, and 32% of participants reported childhood obesity (<10 years). The majority of subjects (76.5%) reported a family history of obesity. Regarding psychiatric comorbidity, 60.6% of the sample had a lifetime diagnosis of mood disorders (i.e., major depressive disorder and bipolar disorder), 55.6% of eating disorders (i.e., BED and bulimia nervosa), and 46.5% of anxiety disorders (i.e., panic disorder, agoraphobia, and social phobia). In addition, 17% of subjects were prescribed mood stabilizers, 26.3% antidepressants, 11.1% benzodiazepines, and 3% antipsychotics at the time of recruitment. Finally, one-third of the sample (33.3%) reported a family history of bipolar disorder, and 16.2% reported a family history of BED.

### 3.2. Comparison of Subjects with Higher (NED+) or Lower (NED−) Negative Emotional Dysregulation (Table 2)

Of the total sample, 49 subjects were classified as NED− (RIPoSt NED score < 70) and 50 as NED+ (RIPoSt NED score ≥ 70). The NED+ and NED− groups did not differ in mean age, proportion of female participants, or mean BMI. In addition, no differences in marital status, education, and employment status were found between the two groups.

**Table 2:** Comparisons between subjects with greater (NED+) or lesser (NED−) negative emotional dysregulation at baseline evaluation.

	NED− (*n* = 49)	NED+ (*n* = 50)	χ2/t	OR (95% C.I.)	*p*
Age (mean, sd)	45.92 (11.13)	45.74 (11.43)	0.079	-	0.937
Female sex (*n*, %)	31 (63.3%)	40 (80.0%)	3.417	2.32 (0.094–5.74)	0.065
BMI (mean, sd)	44.27 (6.21)	45.61 (6.82)	−1.020	-	0.310
Lifetime psychiatric comorbidities (*n*, %)					
Mood disorders Major depressive disorder Bipolar disorder	20 (40.8%)	40 (80%)	15.915	5.80 (2.36–14.22)	<0.001 **
	11 (22.4%)	10 (20.0%)	0.089	0.86 (0.33–2.27)	0.766
	9 (18.4%)	30 (60.0%)	17.966	6.67 (2.66–16.70)	<0.001 **
Eating Disorders Binge-eating disorder Bulimia nervosa	15 (30.6%)	40 (80.0%)	24.447	9.07 (3.61–22.79)	<0.001 **
	13 (26.5%)	37 (74.0%)	22.308	7.88 (3.22–19.29)	<0.001 **
	2 (4.1%)	12 (24.0%)	8.087	7.42 (1.56–35.20)	0.004 **
Anxiety disorders Panic Disorder Agoraphobia Social Phobia	17 (34.7%)	29 (58.0%)	5.404	2.60 (1.15–5.86)	0.020 *
	13 (26.5%)	24 (48.0%)	4.874	2.56 (1.10–5.98)	0.027 *
	10 (20.4%)	13 (26.0%)	0.434	1.37 (0.54–3.51)	0.510
	5 (10.2%)	10 (20.0%)	1.847	2.20 (0.69–6.99)	0.174
ADHD symptomatology (mean, sd)					
WRAADDS_I	3.37 (2.27)	4.62 (2.51)	−2.512	-	<0.014 *
WRAADDS_ED	5.80 (3.15)	9.77 (2.49)	−6.727		<0.001 **
WRAADDS total score	11.78 (5.82)	16.92 (5.79)	−4.408	-	<0.001 **
Impulsivity: BIS-11 (mean, sd)					
Attentional impulsivity	13.27 (2.44)	15.02 (3.27)	−2.657	-	0.010 **
Motor impulsivity	17.08 (3.78)	20.19 (3.77)	−3.638	-	<0.001 **
Non-planning impulsivity	24.48 (4.26)	26.02 (4.56)	−1.534	-	0.129
BIS-11 total score	54.84 (7.54)	61.24 (9.97)	−3.173	-	0.002 **

Abbreviations: ADHD, Attention Deficit/Hyperactivity Disorder; BIS-11, Barratt Impulsiveness Scale, Version 11; BMI, Body Mass Index; WRAADDS, Wender–Reimherr adult attention deficit disorder scale; WRAADDS-I, Wender–Reimherr Adult Attention Deficit Disorder Scale—Inattention; WRAADDS-ED, Wender–Reimherr Adult Attention Deficit Disorder Scale—Emotional dysregulation; * *p* < 0.05; ** significant after Benjamini–Hochberg correction.

As expected, NED+ subjects had a higher rate of comorbidity with bipolar disorder and eating disorders (both BED and bulimia) than NED− subjects. NED+ subjects also had a higher family history of BED (24.0% vs. 8.2%, *p* = 0.032, not significant after Benjamini–Hochberg correction) and a higher prescription of mood stabilizers (32.0% vs. 2.0%, *p* < 0.001). They also showed more frequent comorbidity with anxiety disorders, especially panic disorder, although the latter association did not reach statistical significance after Benjamini–Hochberg correction.

Regarding adult ADHD symptomatology as assessed by the WRAADDS, NED+ patients had higher total scores than NED− patients, and, in particular, higher scores on subscales assessing emotional dysregulation. Finally, NED+ subjects had higher scores on the BIS impulsivity scale, particularly on the attentional and motor impulsivity subscales, while there was no significant difference in the non-planning impulsivity subscale.

In the multivariate logistic regression analysis, we included the following variables: lifetime comorbid bipolar disorder, lifetime comorbid BED, lifetime comorbid bulimia, BIS-11 attentional impulsivity score, BIS-11 motor impulsivity score, WRAADDS_ED score. The logistic regression model was statistically significant (chi-square = 46.806, df = 6, *p* < 0.0001), with 80.6% specificity and 82.1% sensitivity. The model explained 61.9% (Nagelkerke R^2^) of the variance in NED. The clinical features that significantly differentiated NED+ subjects from NED− subjects were lifetime comorbid BED (vs. non-BED, OR = 7.12, 95%C.I. = 1.78–28.62; Wald = 7.653; *p* = 0.006], and WRAADDS_ED score (OR = 1.41, 95%C.I. = 1.11–1.80; Wald = 7.817; *p* = 0.005].

Of the 99 participants initially recruited, 76 underwent bariatric surgery, and 65 of them were re-evaluated at the end of the 1-year follow-up after surgery (i.e., sleeve gastrectomy or Roux-en-Y gastric bypass). Of these subjects, NED+ subjects showed significantly more often an inadequate weight loss than NED− subjects. Specifically, 10 of 28 NED+ subjects (37.5%) had an excess BMI loss of less than 50% one year after surgery compared to 5 of 37 NED− subjects (13.5%) (chi-square = 4.425, *p* = 0.035, OR = 3.55, 95%C.I. = 1.05–12.03).

## 4. Discussion

Our results suggest a significant association between NED and unfavorable outcomes after bariatric surgery in terms of inadequate weight loss at a one-year post-surgical follow-up. Emotional dysregulation represents an impairment in the regulation of affective processes that, among its manifold implications, can lead to disordered eating and facilitate the onset and progression of a ponderal excess. In some individuals with a high level of NED, pathological eating behaviors can become a stereotyped and maladaptive strategy for coping with emotions otherwise difficult to manage (a construct called “emotional eating”) [28,29]. In line with this interpretation, a large body of evidence seems to demonstrate an association between eating disorders, weight cycling, and emotional dysregulation, with a substantial portion of obese individuals showing a particular difficulty in emotional awareness and clarity, a neurodevelopmental fragility known as alexithymia [30,31]. A recent meta-analysis highlighted how disordered eating might be linked to the absence of emotion regulation skills and adaptive strategies, fostering a vicious cycle in eating pathology [32]. The authors concluded that difficulty in emotion regulation did not differ among eating disorders with loss of control (anorexia nervosa binging–purging type, bulimia nervosa, and binge-eating disorder), supporting the transdiagnostic and pathogenetic nature of emotional dysregulation [32,33,34]. An American longitudinal seven-year follow-up study evaluating post-bariatric surgery patients indicates that emotional dysregulation and a greater total affect intensity are associated with poor adherence to post-surgical instructions, resulting in smaller post-surgical weight reduction [35].

A systematic review and meta-analysis highlighted that bariatric patients with ADHD exhibited lower rates of adherence to post-surgical follow-up programs [36]. In addition, these subjects often prefer calorically dense and palatable foods to fulfill the need for immediate gratification and intolerance to delayed gratification. Executive deficits in reward regulation might lead post-bariatric surgery patients with ADHD to perpetuate dysfunctional dietary patterns like overeating, gorging, grazing, and snacking [15,36,37]. Another 12-month follow-up study examined the influence of ADHD symptoms and emotion self-regulation on post-surgical outcomes of 30 patients. Consistent with the results of the present study, an association was found between ADHD symptoms and lower post-intervention weight loss, particularly in subjects with severe emotional dysregulation [38]. These data are intriguing for future assessments regarding the interplay between executive dysfunctions and emotional dysregulation in the pathogenesis and prognosis of bariatric obesity [39,40]. Emotional dysregulation has also been correlated with the partially overlapping construct of cyclothymic–anxious temperament, characterized by marked interpersonal sensitivity, impulsivity, emotional reactivity, and altered regulation of negative affective states, which in turn can lead to disorganized eating [41,42].

Interest in the role of ADHD and emotional dysregulation in obesity is growing, as evidenced by recent literature. In fact, a recent systematic review and meta-analysis highlighted that ADHD is over-represented in a population of bariatric surgery candidates compared to the general population, suggesting that approximately 9% of adult bariatric surgery candidates may be affected. This results in a reduction in quality of life and life expectancy [43].

El Archi et al. assessed the role of emotional dysregulation, alexithymia, and personality dimensions in a sample of 282 bariatric surgery candidates. As expected, emotional dysregulation is a key factor in the association between ADHD and binge eating, with negative emotions acting as a trigger for food-seeking behavior, leading to persistent maladaptive eating behaviors and reduced treatment efficacy [44]. Other studies have also highlighted the association between ADHD and patients undergoing bariatric surgery, emphasizing the role of emotional eating and negative emotional dysregulation [45,46,47].

Assessing sociodemographic factors, a low level of education seems to be correlated with a higher risk of obesity. Our work aligns with the literature, as we observed a lower educational level in our sample compared to the general population in Italy [48]. In our sample, as in almost all studies of obese patient populations, the majority were female, and the average age was between 40 and 50 years [49]. The most prominently highlighted psychiatric comorbidities were mood disorders, eating disorders, and anxiety disorders, with a higher prevalence in NED+ patients [10,22,40].

The long-known association between depression with atypical features and the female sex might partly explain the higher prevalence of obesity in women [50]. Additionally, there is evidence that obesity, when associated with impulsivity and uncontrolled eating, might correlate with bipolarity and mixed features, potentially affecting therapeutic outcomes if left uninvestigated [51,52]. Emotional dysregulation, cyclothymic–anxious-sensitive temperament, according to Akiskal, and hysteroid dysphoria (atypical depression) are part of a pathogenetic and phenomenological continuity [41]. Thus, such mixed/atypical features and “soft bipolarity” signs should be considered as negative prognostic predictors, particularly in female subjects, and deserve careful evaluation for adequate long-term treatment management [50,52,53].

This study presents several limitations. First, the relatively small number of patients included in the sample at the end of the 12-month follow-up may have affected the statistical analyses, with the risk of possible bias in the results. In addition, the use of self-report questionnaires and the retrospective assessment of some clinical variables may have led to reliability problems, as subjects may have underestimated some aspects of their psychiatric status or history in order to access the intervention. Finally, we only collected data on lifetime psychiatric comorbidity, without distinguishing between past and current.

In conclusion, our results suggest that a structured assessment of negative emotional dysregulation in bariatric surgery candidates may be critical in identifying individuals who require closer postoperative follow-up. For these patients, integrated stepped care models should be considered to provide individualized psychiatric interventions after surgery. Unfortunately, the evidence for psychopharmacologic and psychological treatments in this type of individual remains preliminary. Treatment with mood stabilizers or stimulants (e.g., methylphenidate) may be useful in some specific cases to reduce the risk of inadequate weight loss and relapse. Future research is certainly needed on the expansion of current psychiatric interventions, timing of administration, and predictors of response. In addition, further studies are needed to better understand emotion regulation as a psycho(patho)logical mechanism linking eating behaviors to obesity and potentially influencing treatment response and prognosis.

## Data Availability

Data are available on request from the corresponding author.
